# Physical exercise and cognitive training interventions to improve cognition in hemodialysis patients: A systematic review

**DOI:** 10.3389/fpubh.2022.1032076

**Published:** 2022-10-14

**Authors:** Špela Bogataj, Katja Kurnik Mesarič, Maja Pajek, Tanja Petrušič, Jernej Pajek

**Affiliations:** ^1^Department of Nephrology, University Medical Centre Ljubljanag, Ljubljana, Slovenia; ^2^Faculty of Sport, University of Ljubljana, Ljubljana, Slovenia; ^3^Faculty of Education, University of Ljubljana, Ljubljana, Slovenia

**Keywords:** cognitive performance, hemodialysis, physical exercise, cognitive training, cognitive tests, intervention

## Abstract

**Introduction:**

Patients with chronic kidney disease treated with hemodialysis (HD) have lower cognitive abilities compared to the age-matched healthy population. Recently, physical exercise and cognitive training have been presented as possible interventions to improve cognitive abilities both in the general population and in patients with chronic diseases. To date, there is no general overview of the current knowledge on how these interventions affect cognitive abilities in HD patients and what tests are used to measure these effects.

**Methods:**

Three electronic databases were searched for randomized controlled studies of physical exercise or cognitive training interventions that examined effects on cognitive abilities/performance in HD patients.

**Results:**

Six articles were included. All included studies used physical exercise as an intervention, with one study also including tablet-based cognitive training. Four studies included an intradialytic approach and two included a home-based intervention. Intervention lasted. A significant intervention effect was observed in three studies compared with the control condition.

**Conclusion:**

The present review suggests that physical exercise might improve or at least not worsen cognitive performance in HD patients, whereas the effect of cognitive training has not yet been adequately studied. There is a need for more sensitive and specific cognitive tests to adequately measure the effects of interventions in the HD population.

## Introduction

It is well-documented that cognitive deficits cause progression toward dementia (1, 2). This phenomenon is even more pronounced in clinical populations (3). An example of a vulnerable population with an increased incidence of cognitive impairment is patients with kidney disease treated with hemodialysis (HD). The cognitive decline in HD patients is not only the result of underlying and concomitant diseases but can also be attributed to their changed lifestyle after starting HD. These patients have to travel to a dialysis center every other day, where they spend 4–5 h in a sedentary position during a HD procedure. Many reports a post-dialysis burnout and fatigue lasting for up to 24 h post-dialysis (4). As a result, these patients are less physically active and activate their mental functions to a lesser extent. Moreover, in HD patients, diabetes, a common chronic kidney disease (CKD) comorbidity, was significantly associated with larger cognitive impairment (5). HD treatment itself also contributes to a higher risk of developing dementia by causing ischemic stunning of the brain (6). In addition, dementia risk factors such as obesity, depression, and social isolation are common in the HD population.

It was found that only 13% of HD patients have a normal cognitive function (7). Moreover, clinicians usually fail to recognize declining cognitive performance in these patients; therefore, cognitive impairment is critically underestimated and not appropriately treated (8). It has been reported that <5% of all patients with kidney disease with cognitive impairment have been evaluated or received a medical diagnosis (9). Measurement of cognitive function is not currently part of the physical examination and medical history of CKD patients.

Lately, non-pharmacological interventions have been introduced as possible approaches to mitigate cognitive decline and dementia (3). Studies that examined the effect of exercise interventions on cognitive performance showed conflicting results. A systematic review of exercise intervention studies on cognition in older adults did not provide sufficient evidence that exercise affects cognitive performance (10). Another systematic review concluded that physical activity could delay the progression of cognitive decline in the elderly (11). In a recent study, the authors reported the results of a 6-month aerobic exercise intervention in older adults (>60 years) on cognitive function. Compared to control subjects, participants in the training group showed broad improvement in cognitive abilities, including processing speed, episodic memory, executive functions, and updating (12).

In addition to physical activity, cognitive training programs to improve general and specific cognitive domains are being increasingly used in research on cognitive decline. A meta-analysis of 17 controlled interventional trials of computer-assisted cognitive training in subjects with mild cognitive impairment showed a moderate effect on general cognition (13). In community-dwelling older adults, the ACTIVE trial demonstrated long-term retention of a benefit of 10–14 weeks' cognitive training with significant improvement in cognitive abilities and maintenance of functional status after a 10-year period (14).

Despite the fact that there is a plethora of research on physical and cognitive interventions, most of the focus has been on the general population. There is little research addressing the clinical population. In addition, there is no systematic review of the effect of physical exercise and cognitive training that focuses on patients with CKD undergoing HD. Therefore, the aim of this systematic review was to examine the effects of non-pharmacological interventions in the form of cognitive and physical exercise training on different domains of cognitive performance.

## Materials and methods

The review methods and reporting were performed according to the preferred reporting items in systematic review and meta-analyses (PRISMA) guidelines (15).

### Eligibility criteria

The PICOS search tool (participant, intervention, comparison, outcome, and study design) was used to determine keywords (Table 1).

**Table 1:** “PICOS” items (participants, intervention, comparisons, outcomes, study designs) used to select keywords.

**PICOS item**	**Detail**
Participants	Hemodialysis patients
Interventions	Physical exercise training or/and cognitive training
Comparisons	Active or inactive control group
Outcomes	Cognitive performance
Study designs	RCTs

RCT, randomized controlled trial.

Studies were included in the systematic review if they met the following criteria: (a) randomized controlled trials, (b) published in academic journals, (c) written in English, (d) with participants on hemodialysis and (e) studies that included physical exercise or cognitive training interventions with (f) outcome of cognitive performance. Studies were excluded if study population were CKD patients without kidney replacement therapy or patients on peritoneal dialysis, animal studies, and individual case studies.

### Search strategy

**Figure 1:**
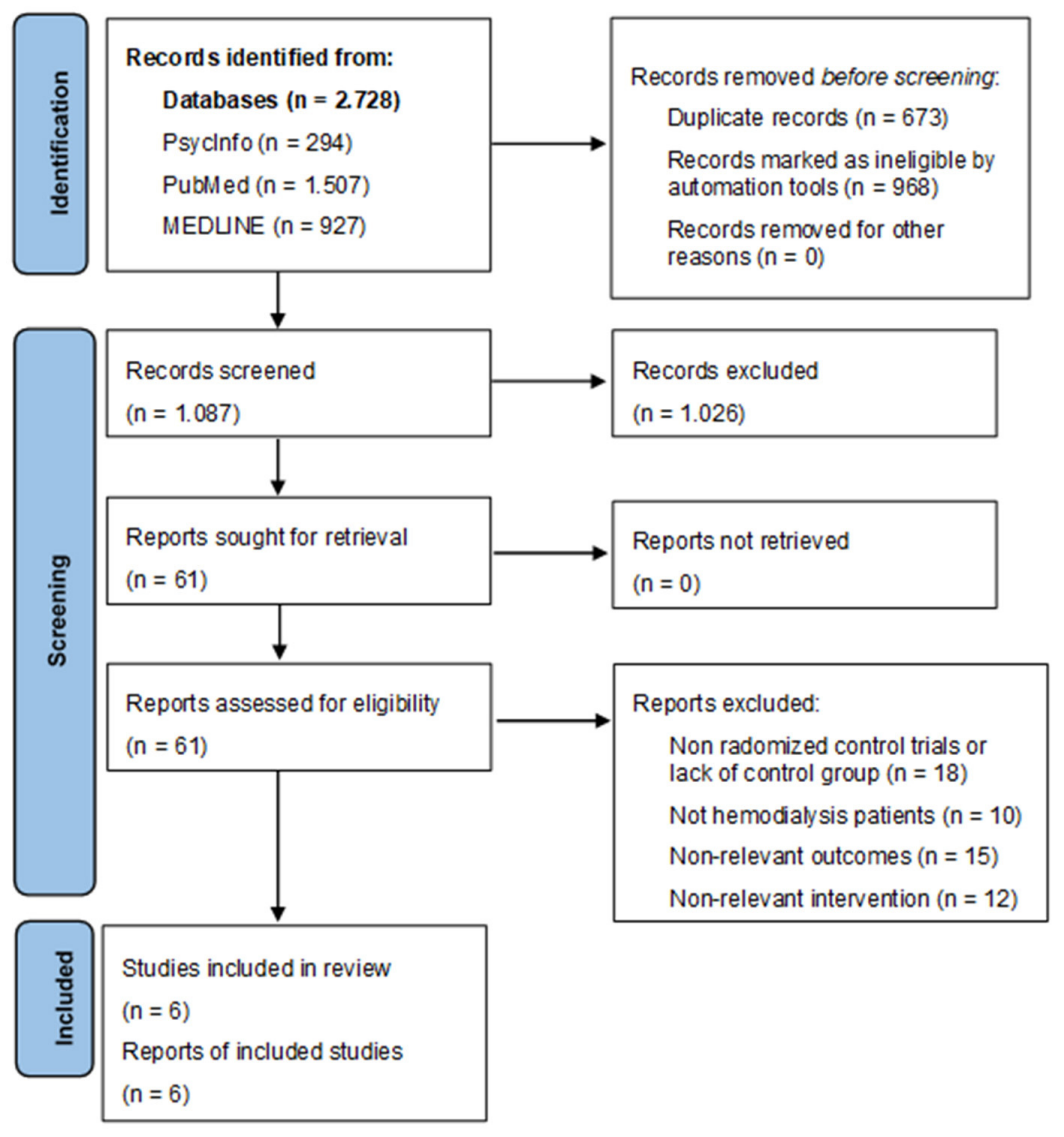
PRISMA flow diagram.

To identify potentially relevant studies, we performed a comprehensive literature search in electronic databases including PsycInfo, PubMed and MEDLINE (Ovid) from the database's inception to the final update in August 2022. Medical subject heading (mesh) terms were used, if available, for a qualitative search of potential studies. Search strategies utilized a combination of key words to represent definitions of hemodialysis, cognitive functioning, physical activity interventions and cognitive training. Terms were combined using the “AND” and “OR” Boolean operator (for the full list of search phrases and terms, see Table 2). To increase the likeliness of including all relevant trials, a backward and forward search were performed by screening the citations and references list of the included studies. A flow diagram of the search is presented in Figure 1.

**Table 2:** Search strategy.

**Literature search**
PsycInfo	Hemodialysis OR haemodialysis AND cognition OR cognitive function OR cognitive performance OR cognitive abilities OR cognitive ability
	Hemodialysis OR haemodialysis AND cognition OR cognitive function OR cognitive performance OR cognitive abilities OR cognitive ability AND intervention
	Hemodialysis OR haemodialysis AND cognitive training
	Hemodialysis OR haemodialysis AND physical activity OR exercise OR fitness OR physical exercise
	Hemodialysis OR haemodialysis AND physical activity OR exercise OR fitness OR physical exercise AND intervention
	Hemodialysis OR haemodialysis AND cognitive intervention
	Renal dialysis AND cognition OR cognitive function OR cognitive performance OR cognitive abilities OR cognitive ability AND intervention
	Renal dialysis AND cognition OR cognitive function OR cognitive performance OR cognitive abilities OR cognitive ability AND intervention AND physical activity OR exercise OR fitness OR physical exercise
	Renal dialysis AND cognition OR cognitive function OR cognitive performance OR cognitive abilities OR cognitive ability
	Renal dialysis AND physical activity OR exercise OR fitness OR physical exercise AND intervention
	Renal dialysis AND cognitive training
Filters	English, academic journals
PubMed	Hemodialysis AND cognition [MeSH]
	Hemodialysis [MeSH] AND cognitive training
	Hemodialysis [MeSH] AND physical activity intervention and cognition [MeSH]
	Hemodialysis [MeSH] AND and exercise [MeSH] AND cognition [MeSH]
	Renal dialysis [MeSH] AND cognition AND intervention
	Renal dialysis [MeSH] AND physical activity AND cognition AND intervention
	Renal dialysis [MeSH] AND cognitive training
	Renal dialysis [MeSH] AND exercise [MeSH]
Filters	English, randomized controlled trials
Medline (OVID)	Renal dialysis AND cognition AND exercise
	Renal dialysis AND cognitive training
	Renal dialysis AND cognitive training OR physical exercise intervention
	Hemodialysis OR renal dialysis AND cognitive training
	Renal dialysis AND physical acitivity intervention OR fitnes intervention OR physical excercise intervention
	Renal dialysis AND cognitive intervention
	Renal dialysis AND cognitive intervention OR physical excercise intervention
	Renal dialysis AND cognit*
Filters	English, academic journals, expand term finder

*Wildcard that finds variant spellings of words.

### Study selection and data collection procedure

Articles from the above databases were checked for duplicates using EndNote X9. Subsequently, all articles were screened using the Rayyan app (16). In the app, two reviewers (ŠB and KKM) independently reviewed the titles and abstracts of each article in a blinded manner. The decision to include an article was made at the research team meeting. Articles that met the inclusion criteria underwent quality assessment.

### Risk of bias assessment

The quality of the included studies was evaluated using the revised Cochrane Collaboration's risk of bias tool (17). The following biases were evaluated: bias arising from the randomization process, bias due to deviations from intended interventions, bias due to missing outcome data, bias in measurement of the outcome, bias in selection of the reported result and overall bias. Risk of bias was assessed as low, some concerns or high for each domain and for overall bias.

## Results

### Study selection

The flow of studies through the review process is reported in Figure 1. Automation tools used in databases were language (English), type of publication (Academic journals) and if possible study type (randomized controlled trial). Duplicate records were removed once the search strategy outputs were combined. Titles and abstracts were screened to identify studies that administered physical activity interventions or cognitive training/interventions to promote cognitive functioning. Full-texts of these articles were read to see whether full inclusion criteria were met. All studies that met inclusion criteria were again screened to determined eligibility for the systematic literature review.

### Quality assessment

**Figure 2:**
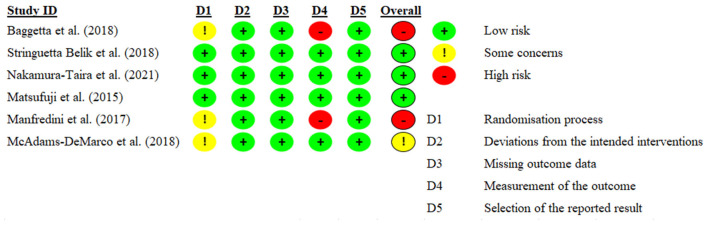
Risk of bias summary.

The bias risks are presented using a risk of bias summary in Figure 2. In the aspects of measurement of outcome, two of the studies were assessed as high risk (18, 19), in the aspects of randomization process three of six included studies were assessed with some concerns (18–20). Three of all included studies were assessed as low risk on all domains and overall (21–23).

### Study characteristics

The six included studies were published between 2015 and 2021. They included a total of 466 HD patients. Two hundred fourteen patients received a physical exercise intervention and seven patients received a cognitive training intervention. The control group, which received standard treatment or stretching exercises, consisted of 245 HD patients. The average age of participants in the included studies ranged from 48 to 75 years. The youngest patients were in the exercise group of the study by McAdams-DeMarco et al. (20), and the oldest were in the control group of the study by Baggetta et al. (18). Two studies did not report the frequency of exercise. The remaining physical activity studies reported that exercise was performed three times per week. The duration of the intervention ranged from 12 to 24 weeks. The characteristics of the included studies are shown in Table 3.

**Table 3:** Characteristics of included studies.

**Study**	**Sample size**	**Age (mean ±SD)**	**Intervention**	**Duration**	**Measures**	**Outcomes**
McAdams-DeMarco et al. (20)	CT = 7 EX = 6 CON = 7	CT = 48.9 ± 12.2 EX = 48.0 ± 7.0 CON = 55.0 ± 9.7	CT = intradialytic cognitive training EX = intradialytic cycling CON = standard care	3x/week 12 weeks	3 MS TMTA TMTB	3 MS (score) mean change from baseline CT: −3.4 (9.2); *p* = 0.24; ES = −0.36 EX: 4.3 (5.4); *p* = 0.17; ES = +0.7 CON: −0.1 (7.0); *p* = 0.96; ES = −0.01 TMTA (s) mean change from baseline CT: −0.2 (14.7); *p* = 0.98; ES = −0.01 EX: −2.5 (9.3); *p* = 0.77; ES = −0.15 CON: 15.0 (25.8); *p* = 0.055; ES = +0.76 TMTB (s) mean change from baseline CT: 0.6 (29.1); *p* = 0.97; ES = +0.02 EX: −8.9 (24.4); *p* = 0.63; ES = −0.46 CON: 47.4 (45.7); *p* = 0.006; ES = +1.1
Manfredini et al. (19)	EX = 104 CON = 123	EX = 63 ± 13 CON = 64 ± 14	EX = walking exercise program CON = standard care	24 weeks	KDQOL-SF cognitive function	Change from baseline (range) EX: +0.3 (−3.2 to 3.8); *p* = 0.87; ES = +0.03 CON: −6.4 (−11.9 to −0.9); *p* = 0.02; ES = −0.44 Changes between groups (range) −6.7 (−13.2 to −0.2); p (EX vs. CON) = 0.04
Matsufuji et al. (21)	EX = 15 CON = 17	EX = 69 ± 11 CON = 69 ± 13	EX = chair stand exercise CON = stretch exercise	EX: 3x/week CON: 1x/week 12 weeks	3MS	Change from baseline (range) EX: 6 (0–17) CON: 2 (−5 to 12) Comparison between groups *p* = 0.40
Nakamura-Taira et al. (22)	EX = 21 CON = 21	EX = 74.9 ± 2.23 CON = 72.57 ± 2.26	EX = intradialytic resistance exercise CON = stretch exercise	3x/week 24 weeks	MoCA	Result at baseline and after 24 weeks EX: 18.45 ± 0.63 (baseline), 18.87 ± 0.71 (at 24 weeks); ES = +0.63 CON: 18.48 ± 0.77 (baseline), 18.09 ± 0.94 (at 24 weeks); ES = −0.45 Comparison between groups SMD = 0.86 95% CI = 0.23–1.5 ES = −0.13 *p* > 0.05
Stringuetta Belik et al. (23)	EX = 15 CON = 15	EX = 50.3 ± 17.24 CON = 57.8 ± 15.01	EX = intradialytic stretch exercises and cycling CON = standard care	3x/week 16 weeks	MMSE	Result at baseline and after 16 weeks
						EX: 24.0 ± 3.0 (baseline), 26.4 ± 2.92 (at 16 weeks); ES = +0.81; *p* < 0.001 CON: 22.4 ± 4.98 (baseline), 23.0 ± 5.09 (at 16 weeks); ES = +0.12; *p* > 0.05 Comparison between groups *p* = 0.023
Baggetta et al. (18)	EX = 53 CON = 62	EX = 73 ± 5 CON = 75 ± 6	EX = walking exercise program CON = standard care	24 weeks	KDQOL-SF cognitive function	Within group change EX: 0.8 (from −4.9 to 6.5); ES = +0.18; *p* = 0.78 CON: −9.6 (from −18.5 to −0.7); ES = −1.74; *p* = 0.04 Between-group difference in change (EX vs. CON) −10.4 (from −21.6 to 0.8); *p* = 0.05

CT, cognitive training group; EX, physical exercise group; CON, control group; 3MS, Modified Mini-Mental State; TMT, Trail-Making Test; KDQOL-SF, Kidney Disease Quality of Life Short Form; MoCA, Montreal Cognitive Assessment; MMSE, Mini-Mental State Examination; ES, Cohens'd effect size; SMD, standardized mean difference; CI, confidence interval; p, statistical significance.

Two included studies examined the effect of a 6-month home-based walking program on self-reported cognitive function as measured by KDQOL-SF (18, 19). In one study, there was a significant increase in cognitive function (19) and in the other study the control group experienced a decline while an experimental group preserved self-reported cognitive function in older dialysis patients (>65 years) (18). An intradialytic exercise program in the form of stretching exercises and cycling significantly improved cognitive performance as measured by the MMSE in the experimental group compared to the standard care control group (23). Contraindicatory, intradialytic resistance exercise (22) and chair stand exercise program (21) showed no significant effect on cognitive ability as measured by MoCA and 3MS. Only one included pilot study examined the effects of cognitive training (20) with 20 HD patients randomly assigned to a cognitive training group (brain games on tablet computers, *n* = 7), an intradialytic cycling group (*n* = 6), or to standard treatment group (*n* = 7). The intervention lasted 3 months and showed a decline in executive functions and psychomotor speed in the control group, whereas the decline was not observed in either the cycling or cognitive training groups.

Regarding the duration of the intervention, in two studies (20, 21), the 12-week intervention did not result in significant improvement in selected cognitive domains. In the remaining studies, the intervention was delivered for 16 weeks or longer and showed either significant improvement (19, 23) or maintenance of cognitive performance compared to the control group (18, 22).

Cognitive performance/ability was assessed using various validated cognitive tests/questionnaires: Modified Mini-Mental State (3MS), Trail-Making Test A and B (TMTA and TMTB), Kidney Disease Quality of Life Short Form (KDQOL-SF), Montreal Cognitive Assessment (MoCA), and Mini-Mental State Examination (MMSE). Brief description of used tests and questionnaires is offered in Table 4.

**Table 4:** Description of cognitive tests/questionnaires.

**Test/questionnaire**	**Description**
Trail-making test A and B (TMTA and TMTB)	TMT is a neuropsychological test that involves visual attention and task switching. It offers information about mental flexibility, visual search speed, speed of processing and executive functioning (24).
Kidney disease quality of life short form (KDQOL-SF)	KDQOL-SF offers disease specific quality of life measure for patients with end-stage renal disease. It includes generic and disease specific components. The scales of KDQOL-SF are: symptoms, effects of kidney disease, burden of kidney disease, work status, cognitive function, quality of social interaction, sexual function, sleep, social support, dialysis staff encouragement and patient satisfaction (25).
Montreal cognitive assessment (MoCA)	MoCA test is screening instrument for mild cognitive disfunction, and it offers information about cognitive domains of attention and concentration, executive functioning, memory, language, orientation, visuospatial abilities, conceptual thinking and orientation (26).
Mini-mental state examination (MMSE)	MMSE is a set of 11 tasks that can be used for assessing cognitive impairment (27). If offers a brief assessment of several cognitive domains: orientation, memory, attention, calculation, language and constructional ability.
Modified mini-mental state (3MS)	3MS is modified MMSE, it includes four additional items, and it extends scoring range. It can be used as a brief cognitive assessment or as a screening test. It offers a brief assessment of following cognitive domains: orientation, attention, concentration, calculation, language abilities, long-term and short-term memory, abstract thinking, and verbal fluency (28).

The MoCA test is a cognitive screening test that has good sensitivity (76.7%) and specificity (78.6%) for assessing cognitive performance in HD patients (29). Another screening test, the MMSE, showed a sensitivity of 55.2% and specificity of 75% (29). The 3MS is a modified version of the MMSE, which showed a sensitivity of 88% and a specificity of 90% as a screening test for dementia in a study of elderly residents (30). In a study by Dobbs and Shergill (31) examining the predictive power of the TMT for driving performance, the TMTA had a sensitivity of 77% and a specificity of 62%, while the TMTB had a sensitivity of 50% and a specificity of 88%. The cognitive domain of the KDQOL-SF had poor sensitivity (range, 28–36%) and modest specificity (range, 77–81%) for identifying poorer memory and executive function in the HD population (32).

## Discussion

In the present review, we highlighted the effects of non-pharmacological interventions (physical exercise or cognitive training) on cognitive performance in HD patients. In addition, we presented and described the cognitive tests used in the included studies. The results of a limited number of studies show that physical exercise may significantly improve cognitive performance or at least mitigate cognitive decline in HD patients. Furthermore, there is insufficient evidence to conclude that cognitive training can attenuate cognitive decline in this population.

Lower cognitive functioning is often seen in HD patients (33–35). It has been documented that impaired cognitive abilities limit the ability to adhere to dialysis activities, make informed decisions, follow food and fluid restrictions, and are a risk factor for mortality in HD patients (36–39). Therefore, the recognition of poor cognitive function is crucial for the implementation of prevention and coping strategies to delay patients' cognitive decline. Furthermore, it is well-known that HD patients have poorer physical function compared to healthy individuals (40) and are mostly physically inactive (41), leading to a decreased quality of life (42). A growing number of randomized controlled trials of exercise training in the HD population show improvement in physical performance (43–46), dialysis symptoms (47), bone mineral density (48, 49), dialysis adequacy (50, 51), and quality of life (52). The highest adherence to physical exercise programs was observed in interventions performed during dialysis (53–55) and these interventions generally appear safe. Notwithstanding the positive effects of physical exercise mentioned above, randomized controlled trials investigating the effects on cognitive performance in HD patients are lacking. However, the limited number of studies included in this review suggests that patients may also benefit in this area.

There are several reasons for the positive association between physical activity and improved cognitive performance. Physical activity has been found to prevent cerebral atrophy or even increase hippocampal volume (56). Furthermore, a recent review found that up to 82% of total brain gray matter volume can be altered by physical activity (57). People in good physical condition can tolerate a higher neuropathological load without suffering cognitive impairment (58). The association between a low cognitive score and high risk or incidence of injury indicates a direct relationship between higher cognitive control and executive function (59, 60). Physical exercise may also have a positive effect on patients' cognitive performance by reducing inflammation and thus improving brain plasticity (61, 62). The results of the present review support the findings of the aforementioned studies in HD patients and contribute to the understanding of the relationship between physical exercise and cognitive performance in this population.

Cognitive training is another non-pharmacological intervention that has received attention in the scientific community. In healthy older adults, cognitive training prevented cognitive decline in executive functions, including working memory, abstracting ability, attentional control, inhibitory control, and verbal reasoning (63–65). Studies investigating cognitive training approaches to combat cognitive decline in HD patients are lacking.

This systematic review has its pitfalls, mainly related to the limitations of the included studies. Limitations include the small number of eligible studies, the small sample size of most included studies leading to low statistical power and possibly associated with potential imbalances in the study groups. The appropriateness of cognitive tests used to measure intervention effects is questionable. The instruments used in the included studies (3MS, MoCA, MMSE, KDQOL-SF) are predominantly screening tests to detect mild cognitive impairment, which are vulnerable to learning effects and may lack sensitivity and specificity (32, 66, 67). Therefore, the aforementioned tests are not the best option to detect the effects of the training interventions presented. Future studies should consider using more sensitive and specific tests instead of using tests that only measure global cognitive performance and are subject to the learning effect. It is proposed to develop a neurocognitive battery to systematically assess various cognitive abilities. Suggested cognitive tests with low learning effect, high sensitivity, validity, and reliability could be the Symbol Digit Modalities Test (SDMT), the Computerized Test of Attentional Performance (TAP), and the Trail Making Test (TMTA and TMTB) (24, 68, 69).

This is the first systematic review to demonstrate the effect of non-pharmacological interventions in the form of physical exercise and cognitive training in HD patients. It also provides insight into the instruments used to measure cognitive performance. These results from a small number of studies suggest that physical exercise training may have a positive effect on cognitive performance in HD patients. The effects of cognitive training or a combination of both approaches should be further investigated (70). Intra-dialysis period provides a unique opportunity to study these effects. Patients could use the time spent during the HD session to replace passive activities with activities that benefit their cognitive status. Research in nephrology has only begun to examine the short-term effects of exercise and cognitive training on cognition. Further studies are needed to replicate these findings and to investigate different strategies to maintain or improve cognitive function not only in HD patients but also in pre-dialysis CKD patients and in transplant recipients. In addition, long-term outcomes such as prevention of dementia should also be investigated. Furthermore, more sensitive and reliable instruments are needed to evaluate the effects of interventions on cognitive performance in this population.

## Data availability statement

The datasets presented in this study can be found in online repositories. The names of the repository/repositories and accession number(s) can be found in the article/supplementary material.

## Author contributions

ŠB designed the search strategy. JP, KM, TP, and MP revised the design. Title and abstract screening was performed by ŠB and KM. Full text screening was performed by ŠB and JP. MP and TP performed data analysis. Quality assessment was performed by KM and ŠB. ŠB drafted the manuscript, which was revised by JP, KM, TP, and MP. All authors approved the final version of the manuscript.

## Funding

The research is funded from ARRS postdoctoral research project Z3-3213 and ARRS research and infrastructure program P3-0323.

## Conflict of interest

The authors declare that the research was conducted in the absence of any commercial or financial relationships that could be construed as a potential conflict of interest.

## Publisher's note

All claims expressed in this article are solely those of the authors and do not necessarily represent those of their affiliated organizations, or those of the publisher, the editors and the reviewers. Any product that may be evaluated in this article, or claim that may be made by its manufacturer, is not guaranteed or endorsed by the publisher.

## References

[1] Messier C Gagnon M. Cognitive decline associated with dementia and type 2 diabetes: the interplay of risk factors. Diabetologia. (2009) 52:2471–4. 10.1007/s00125-009-1533-2 19779694

[2] Pal A Pegwal N Kaur S Mehta N Behari M Sharma R. Deficit in specific cognitive domains associated with dementia in Parkinson's disease. J Clin Neurosci. (2018) 57:116–20. 10.1016/j.jocn.2018.08.016 30150061

[3] Livingston G Huntley J Sommerlad A Ames D Ballard C Banerjee S . Dementia prevention, intervention, and care: 2020 report of the lancet commission. Lancet. (2020) 396:413–46. 10.1016/S0140-6736(20)30367-6 32738937 PMC7392084

[4] Horigan AE. Fatigue in hemodialysis patients: a review of current knowledge. J Pain Symptom Manage. (2012) 44:715–24. 10.1016/J.JPAINSYMMAN.2011.10.015 22743156

[5] Cui L Chen W Yu X Ju C. The relationship between cognitive function and having diabetes in patients treated with hemodialysis. Int J Nurs Sci. (2020) 7:60–5. 10.1016/j.ijnss.2019.12.003 32099861 PMC7031115

[6] McIntyre CW. Haemodialysis-induced myocardial stunning in chronic kidney disease - a new aspect of cardiovascular disease. Blood Purif. (2010) 29:105–10. 10.1159/000245634 20093813

[7] Murray AM. Cognitive impairment in the aging dialysis and chronic kidney disease populations: an occult burden. Adv Chronic Kidney Dis. (2008) 15:123–32. 10.1053/j.ackd.2008.01.010 18334236 PMC2504691

[8] Sehgal AR Grey SF DeOreo PB Whitehouse PJ. Prevalence, recognition, and implications of mental impairment among hemodialysis patients. Am J Kidney Dis. (1997) 30:41–9. 10.1016/S0272-6386(97)90563-1 9214400

[9] Kurella M Mapes D Port FK Chertow GM. Correlates and outcomes of dementia among dialysis patients: the dialysis outcomes and practice patterns study. Nephrol Dialysis Transpl. (2006) 21:2543–8. 10.1093/ndt/gfl275 16751655

[10] Snowden M Steinman L Mochan K Grodstein F Prohaska TR Thurman DJ . Effect of exercise on cognitive performance in community-dwelling older adults: Review of intervention trials and recommendations for public health practice and research. J Am Geriatr Soc. (2011) 59:704–16. 10.1111/j.1532-5415.2011.03323.x 21438861

[11] Carvalho A Rea IM Parimon T Cusack BJ. Physical activity and cognitive function in individuals over 60 years of age: a systematic review. Clin Interv Aging. (2014) 9:661–82. 10.2147/CIA.S55520 24748784 PMC3990369

[12] Jonasson LS Nyberg L Kramer AF Lundquist A Riklund K Boraxbekk CJ. Aerobic exercise intervention, cognitive performance, and brain structure: results from the physical influences on brain in aging (PHIBRA) study. Front Aging Neurosci. (2017) 8:336. 10.3389/fnagi.2016.00336 28149277 PMC5241294

[13] Hill NTM Mowszowski L Naismith SL Chadwick VL Valenzuela M Lampit A. Computerized cognitive training in older adults with mild cognitive impairment or dementia: a systematic review and meta-analysis. Am J Psychiatry. (2017) 174:329–40. 10.1176/appi.ajp.2016.16030360 27838936

[14] Rebok GW Ball K Guey LT Jones RN Kim HY King JW . Ten-year effects of the advanced cognitive training for independent and vital elderly cognitive training trial on cognition and everyday functioning in older adults. J Am Geriatr Soc. (2014) 62:16–24. 10.1111/jgs.12607 24417410 PMC4055506

[15] Page MJ Mckenzie JE Bossuyt PM Boutron I Hoffmann TC Mulrow CD . The PRISMA 2020 statement: an updated guideline for reporting systematic reviews. BMJ. (2021) 372:n71. 10.1136/bmj.n71 33782057 PMC8005924

[16] Ouzzani M Hammady H Fedorowicz Z Elmagarmid A. Rayyan-a web and mobile app for systematic reviews. Syst Rev. (2016) 5:1–10. 10.1186/S13643-016-0384-4/FIGURES/6 27919275 PMC5139140

[17] Higgins JPT Savović J Page MJ Elbers RG Sterne JAC. Assessing risk of bias in a randomized trial. Cochrane Handbook Syst Rev Interv. (2019) 205–28. 10.1002/9781119536604.CH8

[18] Baggetta R D'Arrigo G Torino C Elhafeez SA Manfredini F Mallamaci F . Effect of a home based, low intensity, physical exercise program in older adults dialysis patients: A secondary analysis of the EXCITE trial. BMC Geriatr. (2018) 18:248. 10.1186/s12877-018-0938-5 30342464 PMC6196029

[19] Manfredini F Mallamaci F D'Arrigo G Baggetta R Bolignano D Torino C . Exercise in patients on dialysis: A multicenter, randomized clinical trial. J Am Soc Nephrol. (2017) 28:1259–68. 10.1681/ASN.2016030378 27909047 PMC5373448

[20] McAdams-DeMarco MA Konel J Warsame F Ying H Fernández MG Carlson MC . Intradialytic cognitive and exercise training may preserve cognitive function. Kidney Int Rep. (2018) 3:81–8. 10.1016/j.ekir.2017.08.006 29340317 PMC5762950

[21] Matsufuji S Shoji T Yano Y Tsujimoto Y Kishimoto H Tabata T . Effect of chair stand exercise on activity of daily living: a randomized controlled trial in hemodialysis patients. J Renal Nutr. (2015) 25:17–24. 10.1053/J.JRN.2014.06.010 25194621

[22] Nakamura-Taira N Horikawa N Oka F Igarashi Y Kobayashi S Kato S . Quasi-cluster randomized trial of a six-month low-intensity group-based resistance exercise for hemodialysis patients on depression and cognitive function: a 12-month follow-up. Behav Med. (2021) 2021:741–60. 10.1080/21642850.2021.1966302 34484975 PMC8409964

[23] Stringuetta Belik F Oliveirae Silva VR Braga GP Bazan R Perez Vogt B Costa Teixeira Caramori J . Influence of intradialytic aerobic training in cerebral blood flow and cognitive function in patients with chronic kidney disease: a pilot randomized controlled trial. Nephron. (2018) 140:9–17. 10.1159/000490005 29879707

[24] Arnett JA Labovitz SS. Effect of physical layout in performance of the trail making test. Psychol Assess. (1995) 7:220–1. 10.1037/1040-3590.7.2.220

[25] Hays R Kallich J Mapes D Coons S Amin N. Kidney Disease Quality of Life Short Form (KDQOL-SF), version 1.3: A Manual for Use Scoring. (1997). Available online at: https://www.researchgate.net/profile/Ronald-Hays/publication/274568265_Kidney_Disease_Quality_of_Life_Short_Form_KDQOL-SF_Version_13_A_manual_for_use_and_scoring/links/5cf56b524585153c3db18a19/Kidney-Disease-Quality-of-Life-Short-Form-KDQOL-SF-Version-13-A-manual-for-use-and-scoring.pdf (accessed August 25, 2022).

[26] Nasreddine ZS Phillips NA Bédirian V Charbonneau S Whitehead V Collin I . The montreal cognitive assessment, MoCA: a brief screening tool for mild cognitive impairment. J Am Geriatr Soc. (2005) 53:695–9. 10.1111/j.1532-5415.2005.53221.x 15817019

[27] Pangman VC Sloan J Guse L. An examination of psychometric properties of the mini-mental state examination and the standardized mini-mental state examination: implications for clinical practice. Appl Nurs Res. (2000) 13:209–13. 10.1053/APNR.2000.9231 11078787

[28] Teng E Chui CH. The modified mini-mental state examination (3MS). J Consult Clin Psychol. (1987) 48:314–8. 3611032

[29] Tiffin-Richards FE Costa AS Holschbach B Frank RD Vassiliadou A Krüger T . The montreal cognitive assessment (MoCA) - a sensitive screening instrument for detecting cognitive impairment in chronic hemodialysis patients. PLoS ONE. (2014) 9:e106700. 10.1371/JOURNAL.PONE.0106700 25347578 PMC4209968

[30] Bland RC Newman SC. Mild dementia or cognitive impairment: the modified mini-mental state examination (3MS) as a screen for dementia. Can J Psychiatry. (2001) 46:506–10. 10.1177/070674370104600604 11526806

[31] Dobbs BM Shergill SS. How effective is the trail making test (Parts A and B) in identifying cognitively impaired drivers? Age Ageing. (2013) 42:577–81. 10.1093/AGEING/AFT073 23896609

[32] Sorensen EP Sarnak MJ Tighiouart H Scott T Giang LM Kirkpatrick B . The kidney disease quality of life cognitive function subscale and cognitive performance in maintenance hemodialysis patients. Am J Kidney Dis. (2012) 60:417–26. 10.1053/j.ajkd.2011.12.029 22425261 PMC3547669

[33] Elias MF Elias PK Seliger SL Narsipur SS Dore GA Robbins MA. Chronic kidney disease, creatinine and cognitive functioning. Nephrol Dial Transpl. (2009) 24:2446–52. 10.1093/ndt/gfp107 19297357 PMC2727297

[34] Drew DA Weiner DE Sarnak MJ. Cognitive impairment in CKD: pathophysiology, management, and prevention. Am J Kidney Dis. (2019) 74:782–90. 10.1053/j.ajkd.2019.05.017 31378643 PMC7038648

[35] Ng CZ Tang SC Chan M Tran BX Ho CS Tam WW . A systematic review and meta-analysis of randomized controlled trials of cognitive behavioral therapy for hemodialysis patients with depression. J Psychosom Res. (2019) 126:109834. 10.1016/j.jpsychores.2019.109834 31525637

[36] Drew DA Weiner DE Tighiouart H Scott T Lou K Kantor A . Cognitive function and all-cause mortality in maintenance hemodialysis patients. Am J Kidney Dis. (2015) 65:303–11. 10.1053/j.ajkd.2014.07.009 25240262 PMC4305473

[37] Alosaimi FD Asiri M Alsuwayt S Alotaibi T bin Mugren M Almufarrih A . Psychosocial predictors of nonadherence to medical management among patients on maintenance dialysis </div>. Int J Nephrol Renovasc Dis. (2016) 9:263–72. 10.2147/IJNRD.S121548 27826207 PMC5096770

[38] Angermann S Schier J Baumann M Steubl D Hauser C Lorenz G . Cognitive impairment is associated with mortality in hemodialysis patients. J Alzheimer's Dis. (2018) 66:1529–37. 10.3233/JAD-180767 30412499

[39] Findlay MD Dawson J Dickie DA Forbes KP McGlynn D Quinn T . Investigating the relationship between cerebral blood flow and cognitive function in hemodialysis patients. J Am Soc Nephrol. (2019) 30:147–58. 10.1681/ASN.2018050462 30530658 PMC6317612

[40] Bučar Pajek M Pajek J. Characterization of deficits across the spectrum of motor abilities in dialysis patients and the impact of sarcopenic overweight and obesity. Clin Nutr. (2018) 37:870–7. 10.1016/j.clnu.2017.03.008 28343799

[41] Johansen KL Chertow GM Nga V Mulligan K Carey S Schoenfeld PY . Physical activity levels in patients on hemodialysis and healthy sedentary controls. Kidney Int. (2000) 57:2564–70. 10.1046/j.1523-1755.2000.00116.x 10844626

[42] Sietsema KE Amato A Adler SG Brass EP. Exercise capacity as a predictor of survival among ambulatory patients with end-stage renal disease. Kidney Int. (2004) 65:719–24. 10.1111/j.1523-1755.2004.00411.x 14717947

[43] Segura-Ortí E Kouidi E Lisón JF. Effect of resistance exercise during hemodialysis on physical function and quality of life: randomized controlled trial. Clin Nephrol. (2009) 71:527–37. 10.5414/cnp71527 19473613

[44] Groussard C Rouchon-Isnard M Coutard C Romain F Malardé L Lemoine-Morel S . Beneficial effects of an intradialytic cycling training program in patients with end-stage kidney disease. Appl Physiol Nutr Metabolism. (2015) 40:550–6. 10.1139/apnm-2014-0357 25955722

[45] Frih B Jaafar H Mkacher W Ben Salah Z Hammami M Frih A. The effect of interdialytic combined resistance and aerobic exercise training on health related outcomes in chronic hemodialysis patients: the tunisian randomized controlled study. Front Physiol. (2017) 8:288. 10.3389/fphys.2017.00288 28620308 PMC5449721

[46] Bogataj Š Pajek J Buturović Ponikvar J HadŽić V Pajek M. Kinesiologist-guided functional exercise in addition to intradialytic cycling program in end-stage kidney disease patients: a randomised controlled trial. Sci Rep. (2020) 10:2564–70. 10.1038/s41598-020-62709-1 32235852 PMC7109131

[47] Giannaki CD Hadjigeorgiou GM Karatzaferi C Maridaki MD Koutedakis Y Founta P . A single-blind randomized controlled trial to evaluate the effect of 6 months of progressive aerobic exercise training in patients with uraemic restless legs syndrome. Nephrol Dial Transpl. (2013) 28:2834–40. 10.1093/ndt/gft288 23929523

[48] Liao M-T Liu W-C Lin F-H Huang C-F Chen S-Y Liu C-C . Intradialytic aerobic cycling exercise alleviates inflammation and improves endothelial progenitor cell count and bone density in hemodialysis patients. Medicine. (2016) 95:e4134. 10.1097/MD.0000000000004134 27399127 PMC5058856

[49] Marinho SM Moraes C Barbosa JE dos SM Carraro Eduardo JC . Exercise training alters the bone mineral density of hemodialysis patients. J Strength Cond Res. (2016) 30:2918–23. 10.1519/JSC.0000000000001374 26863587

[50] Parsons TL Toffelmire EB King-VanVlack CE. The effect of an exercise program during hemodialysis on dialysis efficacy, blood pressure and quality of life in end-stage renal disease (ESRD) patients. Clin Nephrol. (2004) 61:261–74. 10.5414/cnp61261 15125032

[51] Bogataj Š Pajek J Buturović Ponikvar J Pajek M. Functional training added to intradialytic cycling lowers low-density lipoprotein cholesterol and improves dialysis adequacy: a randomized controlled trial. BMC Nephrol. (2020) 21:352. 10.1186/s12882-020-02021-2 32811448 PMC7436960

[52] Barcellos FC Santos IS Umpierre D Bohlke M Hallal PC. Effects of exercise in the whole spectrum of chronic kidney disease: a systematic review. Clin Kidney J. (2015) 8:753–65. 10.1093/ckj/sfv099 26613036 PMC4655802

[53] Johansen KL. Exercise in the end-stage renal disease population. J Am Soc Nephrol. (2007) 18:1845–54. 10.1681/ASN.2007010009 17442789

[54] Sheng K Zhang P Chen L Cheng J Wu C Chen J. Intradialytic exercise in hemodialysis patients: a systematic review and meta-analysis. Am J Nephrol. (2014) 40:478–90. 10.1159/000368722 25504020

[55] Bogataj Š Pajek M Buturović Ponikvar J Pajek J. Outcome expectations for exercise and decisional balance questionnaires predict adherence and efficacy of exercise programs in dialysis patients. Int J Environ Res Public Health. (2020) 17:261–74. 10.3390/ijerph17093175 32370202 PMC7246788

[56] Erickson KI Voss MW Prakash RS Basak C Szabo A Chaddock L . Exercise training increases size of hippocampus and improves memory. Proc Natl Acad Sci USA. (2011) 108:3017–22. 10.1073/pnas.1015950108 21282661 PMC3041121

[57] Batouli SAH Saba V. At least eighty percent of brain grey matter is modifiable by physical activity: a review study. Behav Brain Res. (2017) 332:204–17. 10.1016/j.bbr.2017.06.002 28600001

[58] Wallace LMK Theou O Godin J Andrew MK Bennett DA Rockwood K. Investigation of frailty as a moderator of the relationship between neuropathology and dementia in Alzheimer's disease: a cross-sectional analysis of data from the rush memory and aging project. Lancet Neurol. (2019) 18:177–84. 10.1016/S1474-4422(18)30371-5 30663607 PMC11062500

[59] Wilkerson GB. Neurocognitive reaction time predicts lower extremity sprains and strains. Int J Athletic Ther Train. (2012) 17:4–9. 10.1123/ijatt.17.6.4

[60] Herman DC Barth JC. Neuromuscular performance varies with baseline neurocognition: implications for anterior cruciate ligament injury risk and prevention. Orthop J Sports Med. (2015) 3:2325967115S0009. 10.1177/2325967115S00095 27474381 PMC6039105

[61] Loprinzi PD Herod SM Cardinal BJ Noakes TD. Physical activity and the brain: a review of this dynamic, bi-directional relationship. Brain Res. (2013) 1539:95–104. 10.1016/J.BRAINRES.2013.10.004 24120986

[62] Nascimento CMC Pereira JR Pires De Andrade L Garuffi M Ayan C Kerr DS . Physical exercise improves peripheral BDNF levels and cognitive functions in mild cognitive impairment elderly with different bdnf Val66Met genotypes. J Alzheimers Dis. (2015) 43:81–91. 10.3233/JAD-140576 25062900

[63] Mahncke HW Connor BB Appelman J Ahsanuddin ON Hardy JL Wood RA . Memory enhancement in healthy older adults using a brain plasticity-based training program: a randomized, controlled study. Proc Natl Acad Sci USA. (2006) 103:12523–8. 10.1073/pnas.0605194103 16888038 PMC1526649

[64] Levine B Stuss DT Winocur G Binns MA Fahy L Mandic M . Cognitive rehabilitation in the elderly: effects on strategic behavior in relation to goal management. J Int Neuropsychol Soc. (2007) 13:143–52. 10.1017/S1355617707070178 17166313

[65] Anand R Chapman SB Rackley A Keebler M Zientz J Hart J. Gist reasoning training in cognitively normal seniors. Int J Geriatr Psychiatry. (2011) 26:961–8. 10.1002/GPS.2633 20963768

[66] Dong Y Sharma VK Chan BPL Venketasubramanian N Teoh HL Seet RCS . The montreal cognitive assessment (MoCA) is superior to the mini-mental state examination (MMSE) for the detection of vascular cognitive impairment after acute stroke. J Neurol Sci. (2010) 299:15–8. 10.1016/j.jns.2010.08.051 20889166

[67] Lee SH Cho Aj Min Y-K Lee Y-K Jung S. Comparison of the montreal cognitive assessment and the mini-mental state examination as screening tests in hemodialysis patients without symptoms. Ren Fail. (2018) 40:323–30. 10.1080/0886022X.2018.1455589 29633885 PMC6014510

[68] Benedict RHB Deluca J Phillips G LaRocca N Hudson LD Rudick R. Validity of the symbol digit modalities test as a cognition performance outcome measure for multiple sclerosis. Multiple Sclerosis. (2017) 23:721–33. 10.1177/1352458517690821 28206827 PMC5405816

[69] Zimmermann P Fimm B. Test of Attentional Performance 2.3.1. Vera Fimm, Psychologische Testsysteme. (2017). Available online at: https://www.psytest.de/index.php?page=TAP-2-2&hl=en_US (accessed February 1, 2021).

[70] Bogataj Š Trajković N Pajek M Pajek J. Effects of intradialytic cognitive and physical exercise training on cognitive and physical abilities in hemodialysis patients: study protocol for a randomized controlled trial. Front Psychol. (2022) 13:62. 10.3389/FPSYG.2022.835486/BIBTEX 35145465 PMC8821650

